# Health Insurance Type and Outpatient Specialist Care Among Children With Asthma

**DOI:** 10.1001/jamanetworkopen.2024.17319

**Published:** 2024-06-17

**Authors:** Kimberley H. Geissler, Meng-Shiou Shieh, Jerry A. Krishnan, Peter K. Lindenauer, Arlene S. Ash, Sarah L. Goff

**Affiliations:** 1 Department of Healthcare Delivery and Population Sciences, University of Massachusetts Chan Medical School-Baystate, Springfield; 2 Department of Epidemiology and Biostatistics, School of Public Health, University of Illinois Chicago; 3 Division of Pulmonary, Critical Care, Sleep and Allergy, University of Illinois Chicago; 4 Office of Population Health Sciences, University of Illinois Chicago; 5 Department of Medicine, University of Massachusetts Chan Medical School-Baystate, Springfield; 6 Department of Population and Quantitative Health Sciences, University of Massachusetts Chan Medical School, Worcester; 7 Department of Health Promotion and Policy, School of Public Health & Health Sciences, University of Massachusetts Amherst

## Abstract

**Importance:**

Although children with asthma are often successfully treated by primary care clinicians, outpatient specialist care is recommended for those with poorly controlled disease. Little is known about differences in specialist use for asthma among children with Medicaid vs private insurance.

**Objective:**

To examine differences among children with asthma regarding receipt of asthma specialist care by insurance type.

**Design, Setting, and Participants:**

In this cross-sectional study using data from the Massachusetts All Payer Claims Database (APCD) between 2014 to 2020, children with asthma were identified and differences in receipt of outpatient specialist care by whether their insurance was public (Medicaid and the Children’s Health Insurance Program) or private were examined. Eligible participants included children with asthma in 2015 to 2020 aged 2 to 17 years. Data analysis was conducted from January 2023 to April 2024.

**Exposure:**

Medicaid vs private insurance.

**Main Outcomes and Measures:**

The primary outcome was receipt of specialist care (any outpatient visit with a pulmonology, allergy and immunology, or otolaryngology physician). Multivariable logistic regression models estimated differences in receipt of specialist care by insurance type accounting for child and area characteristics including demographics, health status, persistent asthma, calendar year, and zip code characteristics. Additional analyses examined if the associations of specialist care with insurance type varied by asthma persistence and severity, and whether associations varied over time.

**Results:**

Among 198 101 unique children, there were 432 455 child-year observations (186 296 female [43.1%] and 246 159 male [56.9%]; 211 269 aged 5 to 11 years [48.9%]; 82 108 [19.0%] with persistent asthma) including 286 408 (66.2%) that were Medicaid insured and 146 047 (33.8%) that were privately insured. Although persistent asthma was more common among child-year observations with Medicaid vs private insurance (57 381 [20.0%] vs 24 727 [16.9%]), children with Medicaid were less likely to receive specialist care. Overall, 64 239 child-year observations (14.9%) received specialist care, with substantially lower rates for children with Medicaid vs private insurance (34 093 child-year observations [11.9%] vs 30 146 child-year observations [20.6%]). Regression-based estimates confirmed these disparities; children with Medicaid had 55% lower odds of receiving specialist care (adjusted odds ratio, 0.45; 95% CI, 0.43 to 0.47) and a regression-adjusted 9.7 percentage point (95% CI, −10.4 percentage points to −9.1 percentage points) lower rate of receipt of specialist care. Compared with children with private insurance, there was an additional 3.2 percentage point (95% CI, 2.0 percentage points to 4.4 percentage points) deficit for children with Medicaid with persistent asthma.

**Conclusions and Relevance:**

In this cross-sectional study, children with Medicaid were less likely to receive specialist care, with the largest gaps among those with persistent asthma. These findings suggest that closing this care gap may be one approach to addressing ongoing disparities in asthma outcomes.

## Key Points

**Question:**

How common is it for children with asthma to receive at least some outpatient specialist care during a year, and does this differ by their insurance type?

**Findings:**

In this cross-sectional study including 198 101 children, children with asthma who had Medicaid were less likely to receive outpatient specialty care than those with private insurance; the gap was even greater among children with persistent asthma, for whom specialty care may be particularly important.

**Meaning:**

These findings suggest that less specialist care among children with public vs private insurance may contribute to disparities in asthma outcomes.

## Teaser

This cross-sectional study analyzes whether insurance type (private vs public) is associated with differences in receipt of outpatient specialist care among children with asthma.

## Introduction

Asthma is a common chronic condition among children in the US,^1^ causing substantial morbidity, particularly for children in low-income families.^2,3^ Pediatric asthma is usually treated by primary care clinicians,^3,4^ although asthma is a leading reason for children’s emergency department (ED) visits.^3^ Although most children with asthma are successfully treated by primary care clinicians, guidelines recommend an asthma specialist referral when there is poor control despite evidence-based management.^1,5^

Children with Medicaid live in lower-income families, and Medicaid disproportionately insures children from minoritized racial and ethnic groups who are at higher risk of asthma and asthma exacerbations.^6,7,8^ ED and hospital visit rates are substantially higher for children insured with Medicaid,^9^ who are more likely to have poorly controlled asthma.^10^ Differences in asthma specialist care use could contribute to this disparity; however, little is known about use of specialists by children, particularly with respect to comparisons by insurance type or changes over time.^11^ Pediatric specialists are less likely to accept patients with Medicaid than patients with private insurance,^12,13,14^ which may limit access to specialty care.^15,16^ Most studies of specialist use in pediatric asthma have been limited to specific organizations or settings (eg, ED), and include relatively small or nongeneralizable samples. In these studies, asthma specialists are generally underutilized in relation to guideline recommendations.^17^ Studies of differences in utilization by insurance type have had mixed findings, with some showing disparities in specialist utilization^18,19^ and others showing no difference.^20,21^

Primary care clinicians may underuse or overuse specialist referrals, both in general and for asthma specifically. Specialist care for pediatric asthma is more likely to be guideline-concordant than care provided by primary care clinicians, particularly regarding controller medications.^21,22,23,24^ A recent study in Israel^23^ found a specialist visit for children with asthma was followed by increases in controller medications and decreases in rescue medications and oral corticosteroid use, ED visits, and hospitalizations. However, higher specialist use may be associated with increased testing and associated costs^25^ and increased family burden; additionally, overuse of specialists by children who could be adequately treated in a primary care setting may exacerbate specialist supply issues.^26^ Because differences in asthma specialist care use by insurance type could contribute to persistent disparities in outcomes for children with Medicaid, we sought to explore whether differences exist in receipt of asthma specialist care by insurance type.

Using a statewide cohort of children with asthma in Massachusetts, we examined differences in receipt of asthma specialist care by insurance type. Additionally, we examined differences in asthma specialist care for children with and without persistent asthma, and whether there were differential associations with insurance type. Additionally, we examined changes in asthma specialist care by insurance type over time. We hypothesized specialist care would be less common among those with Medicaid compared with those who were privately insured but that differences would be smaller for those with persistent asthma.

## Methods

### Data and Analytic Sample

This cross-sectional study was approved by the University of Massachusetts-Amherst institutional review board with a waiver of informed consent because due to the use of secondary administrative data. The study followed the Strengthening the Reporting of Observational Studies in Epidemiology (STROBE) reporting guideline.^38^ We used the Massachusetts All Payer Claims Database (MA APCD; 2014-2020; version 8 and version 10) to identify children with asthma. The MA APCD includes health insurance enrollment and claims data for most private insurers and Medicaid. We included children aged 2 to 17 years in each year from 2015 to 2020, using 2014 data to identify persistent asthma.

Consistent with prior studies,^27,28^ we identified children with asthma during a year when they met at least 1 of 3 criteria: (1) 1 or more hospital visits (inpatient, ED, and observation stays) with a principal or secondary diagnosis of asthma (any asthma), (2) 2 or more outpatient visits with any asthma diagnosis, or (3) at least 1 outpatient visit with any asthma diagnosis and at least 1 asthma medication (short-acting β_2_-agonist, long-acting β_2_-agonist, inhaled corticosteroid, methylxanthine, mast-cell stabilizer, or a combination).^29,30,31^ We excluded children with emphysema, chronic obstructive pulmonary disease, or cystic fibrosis.^27,32^ We limited the search to child-years with at least 6 months of observed insurance enrollment and Massachusetts residence. Those with missing data or indicators of data errors were excluded.

### Measures

The primary outcome was a binary indicator of receipt of asthma specialist care defined as at least 1 outpatient visit with any asthma diagnosis with a clinician with a taxonomy code indicating any of 3 (general or pediatric) specialties: allergy and immunology, pulmonology, or otolaryngology. The key explanatory variable was enrollment in MassHealth, including Medicaid and the Children’s Health Insurance Program, vs private insurance. For simplicity, we refer to children enrolled in MassHealth as Medicaid insured, as eligibility differs but coverage is similar. Demographics included age category (2-4 years, 5-11 years, and 12-17 years), with categories based on asthma guidelines^33^ and child sex (male and female). To measure health status, we included a spline of the risk score calculated using DxCG Intelligence software version 6.1 (Cotiviti) as a summary morbidity score based on diagnosis codes.^34^ To determine asthma persistence and severity, we defined persistent asthma during a year based on the Healthcare Effectiveness Data and Information Set (HEDIS) metric; it included children in the cohort enrolled for 2 continuous years who met at least 1 of these criteria in the current and prior year: (1) at least 1 inpatient visit with a principal asthma diagnosis, (2) at least 1 ED visit with a principal asthma diagnosis, (3) at least 4 outpatient or hospital observation visits on different dates with any asthma diagnosis and at least 2 asthma medication fills on different dates, or (4) at least 4 asthma medication fills on different dates.^29,35^ To examine differences in resource availability by 5-digit zip code, we used the composite Structural Racism Effect Index that summarizes multiple dimensions of area-level sources of deprivation andabundance^36^; we also included indicators of county level clinician supply.^37^

### Statistical Analysis

We first provided descriptive statistics, showing differences in characteristics between those with Medicaid and private insurance using χ^2^ and *t* tests. We then estimated differences in receipt of specialist care by insurance type, overall and by specialty, and conducted statistical comparisons using χ^2^ tests.

We estimated logistic models, first unadjusted, and then adjusted for child and community-level factors. In adjusted models, we accounted for child demographics (age category and sex), risk score (spline), persistent asthma (yes or no), indicator variables for each calendar year, the Structural Racism Effect Index, and clinician supply (nurse practitioners, physician assistant, family medicine physicians, and pediatricians per 100 000 population). We reported estimated probabilities and average marginal effects for differences between Medicaid and private insurance.

To examine whether the association of receipt of specialist care with insurance type varied for those with persistent asthma, we estimated a model with an interaction term between Medicaid and persistent asthma. To determine whether the association of receipt of specialist care with insurance type changed over time, we estimated a model with interaction terms between Medicaid and calendar year.

We also conducted sensitivity analyses. First, because overall visit rates for asthma may vary by insurance type, we conducted an analysis limited to children with at least 1 outpatient visit with any asthma diagnosis code in the year. Second, because the persistent asthma indicator is utilization-based and thus may be partially confounded with receipt of specialist care, we examined the sensitivity of our results excluding this indicator. Third, because changes over time may differ for children with persistent asthma, we repeated our analysis examining associations of receipt of specialist care with insurance type over time for children with persistent asthma.

To account for correlation of error terms by geographic area and for multiple child-year observations for the same child, standard errors in all models were 2-way, clustered by 5-digit patient zip code and by child. The delta method was used to estimate standard errors for marginal effects and estimated probabilities. A 2-sided α of .05 was considered significant. Statistical analyses were conducted in SAS version 9.1 (SAS Institute) and Stata-MP version 18.0 (StataCorp). Data analysis was conducted from January 2023 to April 2024.

## Results

Among 198 101 unique children, there were 432 455 child-year observations (186 296 female [43.1%] and 246 159 male [56.9%]; 211 269 aged 5 to 11 years [48.9%]; 82 108 [19.0%] with persistent asthma). Of these child-year observations, 286 408 (66.2%) had Medicaid and 146 047 (33.8%) had private insurance (Table 1 and eFigure 1 in Supplement 1). Those with Medicaid were younger, had higher risk scores (more comorbid illness), and lived in areas with higher Structural Racism Equity Index scores (indicating lower-resourced areas). Rates of persistent asthma were 57 381 (20.0%) among those with Medicaid and 24 727 (16.9%) among those with private insurance. All differences were statistically significant. Each child contributed a mean (SD) of 2.18 (1.43) child-years to the analysis (eTable 1 in Supplement 1).

**Table 1.** Descriptive Statistics for Children With Asthma by Insurance Type, 2015-2020

Variable	Child-year observations by insurance type, No (%)			*P* value
	Overall (N = 432 455)	Medicaid (n = 286 408)	Private insurance (n = 146 047)	
Any outpatient asthma specialist visit	64 239 (14.9)	34 093 (11.9)	30 146 (20.6)	<.001
Child age, y				
2-4	61 975 (14.3)	43 526 (15.2)	18 449 (12.6)	<.001
5-11	211 269 (48.9)	145 469 (50.7)	65 800 (45.0)	
12-17	159 211 (36.8)	97 413 (34.0)	61 798 (42.3)	
Child sex				
Female	186 296 (43.1)	123 932 (43.3)	62 364 (42.7)	<.001
Male	246 159 (56.9)	162 476 (56.7)	83 683 (57.3)	
Insurance type				
Medicaid	286 408 (66.2)	286 408 (100)	0	NA
Private	146 047 (33.8)	0	146 047 (100)	
Comorbidity risk score, mean (SD)	1.22 (2.90)	1.29 (3.03)	1.09 (2.62)	<.001
Persistent asthma	82 108 (19.0)	57 381 (20.0)	24 727 (16.9)	<.001
Structural Racism Effect Index, mean (SD)^a^	−0.48 (1.20)	−0.21 (1.20)	−0.99 (1.03)	<.001
Clinician supply variables per 100 000 population, mean (SD)				
Nurse practitioners	104.93 (62.12)	110.31 (67.00)	94.37 (49.54)	<.001
Physician assistants	43.66 (26.42)	47.22 (27.73)	36.67 (22.02)	<.001
Family practice physicians	22.44 (8.15)	22.09 (8.41)	23.13 (7.54)	<.001
Pediatricians	24.58 (9.61)	24.16 (9.97)	25.41 (8.82)	<.001

Abbreviation: NA, not applicable.

^a^ Structural Racism Effect Index is a composite index of resource availability, which was explicitly constructed using a structural racism framework. The index is normed to 0, with an SD of 1 at the national level. Negative numbers indicate more resource-rich areas while positive numbers indicate less resource-rich areas.

Of the full cohort, 64 239 (14.9%) received asthma specialist care, including 34 093 (11.9%) of those with Medicaid vs 30 146 (20.6%) of those with private insurance (Table 2). The most common specialist type was allergy and immunology, the use of which was less than one-half as much among child-years with Medicaid (20 265 child-years [7.1%]) as among child-years with private insurance (23 199 child-years [15.9%]). Among those with persistent asthma, 26 426 (32.2%) received asthma specialist care compared with 37 813 (10.8%) among those without persistent asthma.

**Table 2.** Types of Asthma Specialist Care Received by Children With Asthma by Insurance Type

Variable	Child-year observations by insurance type, No./Total No. (%)			*P* value
	Overall	Medicaid	Private insurance	
All children with asthma				
Any outpatient specialist visit with any asthma diagnosis	64 239/432 455 (14.9)	34 093/286 408 (11.9)	30 146/146 047 (20.6)	<.001
Any outpatient visit with any asthma diagnosis with a specialist				
Allergy and immunology	43 464/432 455 (10.1)	20 265/286 408 (7.1)	23 199/146 047 (15.9)	<.001
Pulmonology	21 905/432 455 (5.1)	14 445/286 408 (5.0)	7460/146 047 (5.1)	.36
Otolaryngology	1660/432 455 (0.4)	1044/286 408 (0.4)	616/146 047 (0.4)	.004
Children with asthma with at least 1 outpatient visit with asthma diagnosis^a^				
Any outpatient specialist visit with any asthma diagnosis	64 239/290 523 (22.1)	34 093/186 704 (18.3)	30 146/103 819 (29.0)	<.001
Any outpatient visit with any asthma diagnosis with a specialist				
Allergy and immunology	43 464/290 523 (15.0)	20 265/186 704 (10.9)	23 199/103 819 (22.3)	<.001
Pulmonology	21 905/290 523 (7.5)	14 445/186 704 (7.7)	7460/103 819 (7.2)	<.001
Otolaryngology	1660/290 523 (0.6)	1044/186 704 (0.6)	616/103 819 (0.6)	.24

^a^ Sample was limited to children with at least 1 outpatient evaluation and management visit with any asthma diagnosis code in the calendar year.

**Figure 1.**
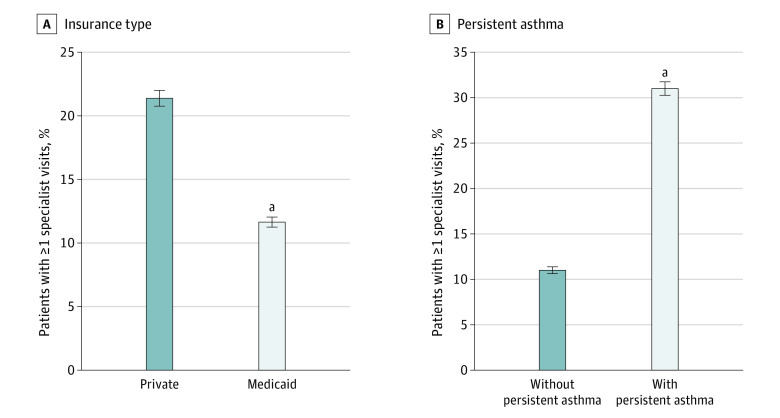
Regression-Adjusted Estimated Probabilities of Receipt of Asthma Specialist Care Based on 432 455 child-year observations. ^a^Indicates difference between private insurance and Medicaid (A) or difference between those with and without persistent asthma (B; statistically significant at *P* < .05). Estimated probabilities were calculated from a logistic regression adjusting for child demographics (age category and sex), risk score spline, an indicator of persistent asthma, indicator variables for calendar year, the Structural Racism Effect Index, and county level indicators of clinician supply (nurse practitioners, physician assistants, family medicine physicians, and pediatricians per 100 000 population). Regression in panel B also includes a control for insurance type (Medicaid vs private).

In both unadjusted and regression-adjusted analyses, those with Medicaid were significantly less likely to have received asthma specialist care than those who were privately insured. Unadjusted analyses showed that compared with those who were privately insured, those with Medicaid had 48% lower odds of receiving specialist care (odds ratio [OR], 0.52; 95% CI, 0.50 to 0.54), or an 8.7 percentage point lower estimated probability of receiving specialist care (95% CI, −9.4 percentage points to −8.1 percentage points) (eTable 2 and eFigure 2 in Supplement 1). After accounting for child age, sex, comorbidity, persistent asthma, year, and community characteristics, those with Medicaid were 9.7 percentage points (95% CI, −10.4 percentage points to −9.1 percentage points) less likely to receive asthma specialist care than those who were privately insured (estimated rates of 11.6% for those with Medicaid vs 21.4% for private insurance; 95% CI of difference, −10.3 percentage points to −9.1 percentage points) (Figure 1). Those with Medicaid insurance had 55% lower odds of asthma specialist use than those with private insurance (adjusted OR [aOR], 0.45; 95% CI, 0.43 to 0.47) (Table 3 and eTable 2 in Supplement 1). This model also showed those with persistent asthma were 20.2 percentage points more likely to receive asthma specialist care than those without (95% CI, 19.5 percentage points to 20.9 percentage points) (Figure 1B and eFigure 2 in Supplement 1). Those with persistent asthma also had 296% greater odds of having received asthma specialist care (aOR, 3.96; 95% CI, 3.80 to 4.14). When we included an interaction term of persistent asthma and Medicaid, we found the greater receipt of asthma specialist care among children with persistent asthma was further exaggerated by 3.2 percentage points (95% CI, 2.0 percentage points to 4.4 percentage points) in children with private insurance compared with Medicaid insurance (receipt in Medicaid vs private insurance, −24.0 percentage points [95% CI, −25.0 percentage points to −23.0 percentage points] among those with persistent asthma vs −20.8 percentage points [95% CI, −21.6 percentage points to −20.0 percentage points] for those without persistent asthma).

**Table 3.** Full Logistic Regression Results

Outcome: receipt of any outpatient specialist care	aOR (95% CI)
Insurance	
Private	1 [Reference]
Medicaid	0.45 (0.43-0.47)
Persistent asthma	3.96 (3.80-4.14)
Sex	
Male	1 [Reference]
Female	1.00 (0.97-1.02)
Age, y	
2-4	1 [Reference]
5-11	1.10 (1.06-1.15)
12-17	0.90 (0.85-0.95)
Comorbidity risk score (spline)	
0-1 (1)	4.35 (4.08-4.64)
1-2 (2)	0.16 (0.15-0.18)
2-4 (3)	1.63 (1.53-1.74)
5-10 (4)	0.87 (0.83-0.91)
≥10 (5)	0.99 (0.96-1.02)
Structural Racism Effect Index	0.92 (0.89-0.96)
Clinician supply (rates per 100 000 population)	
Nurse practitioners	0.99 (0.99-1.00)
Physician assistants	1.01 (1.01-1.01)
Family practice physicians	1.00 (0.9969-1.01)
Pediatricians	1.00 (0.99-1.00)
Calendar year	
2015	1 [Reference]
2016	0.97 (0.94-0.99)
2017	0.91 (0.89-0.94)
2018	0.89 (0.86-0.92)
2019	0.92 (0.88-0.95)
2020	1.00 (0.96-1.04)
Constant^a^	0.11 (0.10-0.13)
Child-year observations, No.	432 455

Abbreviation: aOR, adjusted odds ratio.

^a^ Constant estimates baseline odds; 95% CIs have been calculated using standard errors adjusted for 2-way clustering by child 5-digit zip code and by child.

**Figure 2.**
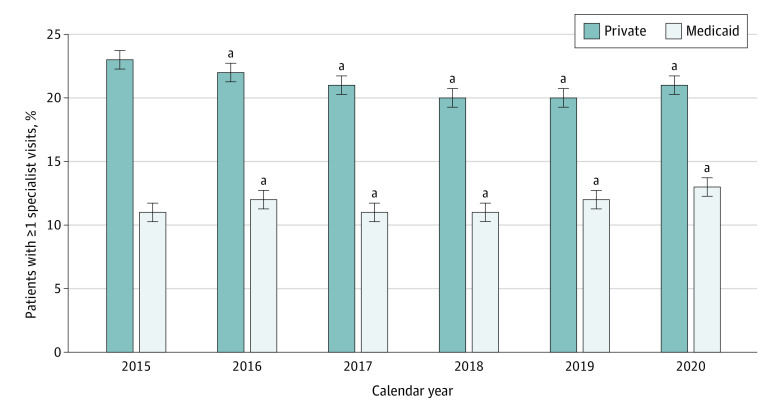
Regression-Adjusted Differences in Receipt of Any Asthma Specialist Care Over Time by Insurance Type Based on 432 455 child-year observations. ^a^Indicates difference from 2015 of same insurance type and is statistically significant at *P* < .05. Estimated probabilities were calculated from logistic regressions adjusting for child demographics (age category and sex), risk score spline, an indicator of persistent asthma, indicator variables for calendar year, the Structural Racism Effect Index, and county level indicators of clinician supply (nurse practitioners, physician assistants, family medicine physicians, and pediatricians per 100 000 population).

As measured in analyses including an interaction term between calendar year indicators and Medicaid, we found the gap in receipt of asthma specialist care between children with Medicaid and private insurance narrowed slightly over time. This reduction in the gap between insurance types over time was due to small increases in receipt of specialist care among children with Medicaid with concomitant decreases in receipt of specialist care among children with private insurance (Figure 2 and eFigure 3 in Supplement 1).

The results of sensitivity analyses showed that our finding of lower receipt of specialist care among children with Medicaid were similar in other specifications. When limiting the sample to child-year observations that had at least 1 outpatient visit with any asthma diagnosis in the calendar year, we found similar results as our main analysis. Children with Medicaid were 12.3 percentage points less likely to have a specialist visit than children with private insurance (estimated rates of 17.8% for those with Medicaid vs 30.1% for those with private insurance; 95% CI of difference, −13.1 percentage points to −11.5 percentage points; aOR, 0.47; 95% CI, 0.45 to 0.49) after accounting for child and community characteristics (eTable 2 in Supplement 1). When excluding the indicator of persistent asthma, findings were similar in direction and magnitude (eTable 2 in Supplement 1). Changes over time by insurance type were similar in direction for those with persistent asthma, but not all differences were statistically significant (eFigure 4 in Supplement 1).

## Discussion

In this cross-sectional study, we found striking disparities in the receipt of specialist care based on insurance type, with children with Medicaid having substantially lower rates of asthma specialist care than those with private insurance, despite children with Medicaid having higher rates of persistent asthma. Contrary to expectations, disparities in specialist care by insurance type were even more striking in children with persistent asthma. Differences in receipt of specialist care were mostly associated with differences in visits with allergy and immunology physicians, with limited but still statistically significant differences in visits with pulmonologists or otolaryngologists.

Although adequate access to specialty care for children with asthma may improve outcomes, including reduction in use of ED and hospital care,^3^ the optimal rate of specialist use for children with asthma is unknown. Prior research^39^ has shown that pediatric primary care clinicians who have higher specialist referral rates were more likely to provide care in line with guideline recommendations. In the US, national asthma guidelines^40^ recommend a specialist referral in patients with persistent asthma if step 3 medications (medium dose inhaled corticosteroids) in children aged 0 to 4 years or step 4 medications (medium dose inhaled corticosteroids plus long-acting β_2_-agonists) in children aged 5 years and older are required, or if further evaluation of allergic asthma for immunotherapy is being considered. The Global Initiative for Asthma^41^ also recommends specialist care in cases of difficulty with asthma diagnosis, frequent health care utilization, food allergy or anaphylaxis, or in patients with multiple chronic conditions.^40,42^ Additional studies are needed to examine if the differential use of asthma specialists are uniform across these subsets of children with asthma.^39^

Our results are consistent with previous studies showing lower utilization of asthma specialists by children with Medicaid.^18,19^ However, these lower rates of specialist care are not always observed, particularly among children surveyed at ED presentation; several studies analyzing a specific subset of children from racial and ethnic minoritized groups presenting to an urban ED found no differences in prior specialist use by insurance type.^21^ Although we did not examine mechanisms by which this disparity occurs, it could be consistent with studies suggesting a lower acceptance of Medicaid by pediatric specialists.^12,13,14^

We found that the gap between children with Medicaid vs private insurance narrowed slightly over time, with reductions in specialist use by children with private insurance along with slight increases in use by children with Medicaid, which was true overall and for children with persistent asthma. Additionally, over the last decade, specialty drugs (ie, biologics) are increasingly available for treatment of moderate to severe asthma with different biological phenotypes.^43,44^ These medications are almost exclusively prescribed by specialists,^45,46^ so ensuring access to specialists is key to ensuring equitable identification of eligibility and treatment with these agents. Although uncommonly used in the pediatric setting, studies^47^ have shown substantial disparities in their use among children with private vs public insurance. Thus, ensuring that children with Medicaid have adequate specialist access may be an integral component of reducing disparities in asthma control by insurance type for those with severe asthma or poor asthma control.^48^

### Limitations

There were several limitations. First, the analysis was limited to Massachusetts, which may not be nationally generalizable. However, asthma prevalence and high rates of poorly controlled asthma among children in Massachusetts is similar to national averages.^49^ In Massachusetts, and nationally, more than one-third of children are Medicaid insured.^50^ Massachusetts introduced primary care focused Medicaid Accountable Care Organizations in 2018. We have not explicitly examined associations of this program with specialist care, but we found limited changes over time in receipt of specialist care for the Medicaid population. Second, we cannot observe referrals to specialists (from primary care clinicians or otherwise), and thus used completed specialist visits as a proxy for referrals. Thus, we cannot observe whether children with Medicaid vs private insurance have similar referral rates but lower rates of completed referrals, or whether there are differential referral rates, or both. Prior research^17^ showed that among parents with children seeking ED care for asthma, the majority of whom met referral guidelines, about 80% of parents not seeing an asthma specialist expressed a desire to see one; parents attributed lack of specialist care to a perceived lack of necessity by their primary care clinician. Future research should address this to better understand mechanisms underlying our findings. Third, we cannot observe asthma symptom frequency or control based on the dose of medications, so we relied on HEDIS indicators of persistent asthma. We also did not distinguish the setting in which or clinician type by whom an asthma diagnosis was made. Fourth, the ideal rate of specialist care among children with asthma is unknown, and thus our results should be interpreted with some caution. However, we found similar results of lower specialist use among children with Medicaid than with private insurance when limiting to children with at least 1 outpatient asthma visit and found differentially lower use of specialists for children with Medicaid with persistent asthma (for whom specialist care is more likely to be recommended) than children with private insurance with persistent asthma. Fifth, although the dataset had full reporting for children with Medicaid, reporting by private insurers starting in 2016 was limited for self-insured employer plans.^51^ Thus, our overall rates should not be interpreted as representative of the Massachusetts population. Additionally, rates of identified asthma were lower in 2020 due to the COVID-19 pandemic^52,53^; we used indicator variables for each year in our models to account for this, but future research should examine whether there are further changes in use of specialist care for pediatric asthma in a post–COVID-19 health care landscape. Sixth, the MA APCD does not contain information on child race or ethnicity, and thus we were unable to examine whether differences exist in receipt of specialist care by race and ethnicity, a key next step for research.

## Conclusions

Novel approaches to integrating of specialty care into primary care, such as electronic consultation, is an increasingly used innovation designed to deliver appropriate specialty care to children.^54^ Other experiments such as primary care oriented interdisciplinary asthma clinics may improve quality of care and outcomes for children with all insurance types.^55^ Our findings suggest children with asthma who have Medicaid receive specialty care at rates significantly lower than children with private insurance, and that this gap is exaggerated further in children with persistent asthma. Ongoing attention to disparities in asthma care quality and outcomes by insurance type, and the contributions of specialist consultation, is warranted.

## Notes

Supplement 1. **eFigure 1.** Construction of Analytic Sample **eTable 1.** Distribution of Children by Number of Years Included in Analytic Sample **eTable 2.** Full Regression Results for Unadjusted and Regression Models, Including Sensitivity Analyses **eFigure 2.** Unadjusted Probabilities of Receipt of Asthma Specialist Care **eFigure 3.** Unadjusted Probabilities of Receipt of Asthma Specialist Care Over Time by Insurance Type **eFigure 4.** Regression-Adjusted Predicted Probabilities of Receipt of Asthma Specialist Care Over Time Limited to Children With Persistent Asthma by Insurance Type

Supplement 2.
Data Sharing Statement
