# Supplementary material for: Health Insurance Type and Outpatient Specialist Care Among Children With Asthma
Source: JAMA Netw Open. 2024 Jun 17;7(6):e2417319. doi: 10.1001/jamanetworkopen.2024.17319 (PMC11184461; doi:10.1001/jamanetworkopen.2024.17319)
Supplement: Supplement 1. — eFigure 1. Construction of Analytic Sample eTable 1. Distribution of Children by Number of Years Included in Analytic Sample eTable 2. Full Regression Results for Unadjusted and Regression Models, Including Sensitivity Analyses eFigure 2. Unadjusted Probabilities of Receipt of Asthma Specialist Care eFigure 3. Unadjusted Probabilities of Receipt of Asthma Specialist Care Over Time by Insurance Type eFigure 4. Regression-Adjusted Predicted Probabilities of Receipt of Asthma Specialist Care Over Time Limited to Children With Persistent Asthma by Insurance Type [file jamanetwopen-e2417319-s001.pdf]

Supplemental Online Content

Geissler KH, Shieh MS, Krishnan, JA, Lindenauer, PK, Ash AS, Goff SL. Health insurance type and outpatient specialist care among children with asthma. *JAMA Netw. Open*. 2024;7(6):e2417319. doi:10.1001/jamanetworkopen.2024.17319

eFigure 1. Construction of Analytic Sample
eTable 1. Distribution of Children by Number of Years Included in Analytic Sample
eTable 2. Full Regression Results for Unadjusted and Regression Models Including Sensitivity Analyses
eFigure 2. Unadjusted Probabilities of Receipt of Asthma Specialist Care
eFigure 3. Unadjusted Probabilities of Receipt Of Asthma Specialist Care Over Time by Insurance Type
eFigure 4. Regression-Adjusted Predicted Probabilities of Receipt of Asthma Specialist Care Over Time Limited to Children With Persistent Asthma by Insurance Type

This supplemental material has been provided by the authors to give readers additional information about their work.

© 2024 Geissler KH et al. *JAMA Network Open.*

eFigure 1. Construction of Analytic Sample

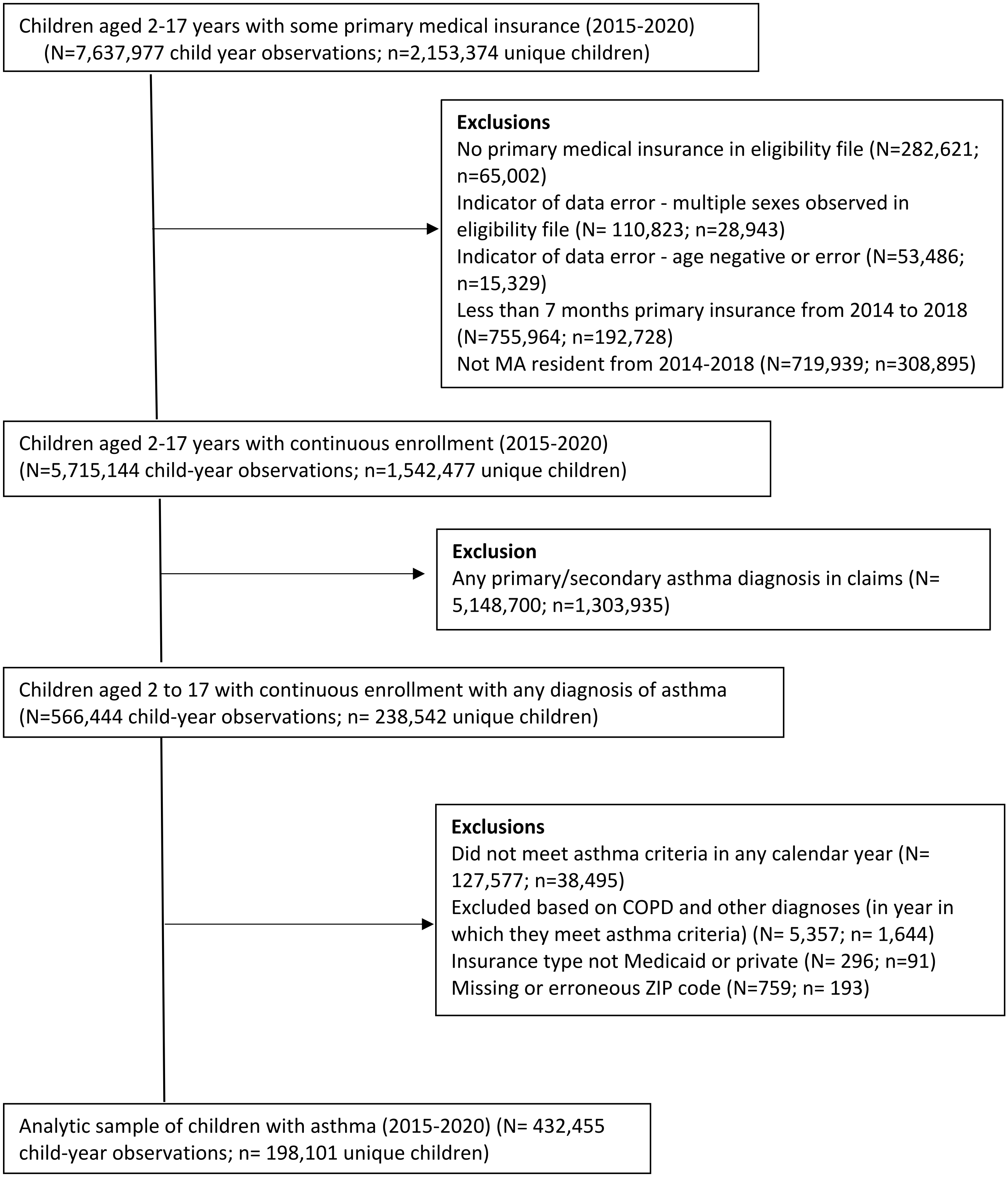

© 2024 Geissler KH et al. *JAMA Network Open.*

eTable 1. Distribution of Children by Number of Years Included in Analytic Sample

Number of Years Included	Unique Children (N=198,101) N	%
1	91,210	46.0
2	42,521	21.5
3	27,200	13.7
4	18,377	9.3
5	11,663	5.9
6	7,130	3.6

Note: This table shows the number of child-years for each unique child included in the final analytic sample. For example, 3.6% of unique children are observed, meet insurance and residence requirements, and meet the asthma criteria in all six years of the data.

© 2024 Geissler KH et al. *JAMA Network Open.*

eTable 2. Full Regression Results for Unadjusted and Regression Models Including Sensitivity Analyses

	Unadjusted model		Main model		Without control for persistent asthma		Restricted to those with any outpatient asthma visit[a]	
	Regression 1		Regression 2		Regression 3		Regression 4	
**Outcome: Receipt of any outpatient specialist care**	**Adj Odds Ratio**	**95% CI**	**Adj Odds Ratio**	**95% CI**	**Adj Odds Ratio**	**95% CI**	**Adj Odds Ratio**	**95% CI**
Medicaid (ref= private insurance)	0.5195	(0.4966,0.5434)	0.4505	(0.4289,0.4732)	0.4857	(0.4630, 0.5096)	0.4672	(0.4452, 0.4902)
Persistent asthma			3.9632	(3.7975,4.1360)	NA	NA	3.3182	(3.1938, 3.4474)
Sex is female (ref=male)			0.9997	(0.9733,1.0268)	0.9722	(0.9463, 0.9987)	0.9861	(0.9595, 1.0135)
Age category (reference = 2-4 years)								
5-11 years			1.1034	(1.0608,1.1477)	1.3150	(1.2679, 1.3638)	1.2014	(1.1517, 1.2531)
12-17 years			0.8999	(0.8537,0.9485)	0.9875	(0.9373, 1.0404)	1.1376	(1.0750, 1.2039)
DxCG comorbidity risk score (spline)								
spline 1 (0-1)			4.3535	(4.0845,4.6402)	4.7619	(4.4758, 5.0664)	3.9123	(3.6544, 4.1884)
spline 2 (1-2)			0.1610	(0.1459,0.1777)	0.1526	(0.1386, 0.1680)	0.1806	(0.1626, 0.2007)
spline 3 (2-5)			1.6333	(1.5294,1.7442)	1.5987	(1.4993, 1.7047)	1.6647	(1.5523, 1.7852)
spline 4 (5-10)			0.8705	(0.8335,0.9092)	0.8557	(0.8200, 0.8930)	0.8571	(0.8182, 0.8978)
spline 5 (10+)			0.9908	(0.9635,1.0190)	0.9990	(0.9721, 1.0267)	0.9988	(0.9687, 1.0298)
Structural racism effect index			0.9255	(0.8926,0.9596)	0.9316	(0.8996, 0.9646)	0.9423	(0.9081, 0.9779)
Clinician supply (rates per 100,000 population)								
Nurse Practitioners			0.9938	(0.9924,0.9953)	0.9942	(0.9928, 0.9956)	0.9928	(0.9914, 0.9943)
Physician Assistants			1.0087	(1.0056,1.0117)	1.0089	(1.0059, 1.0120)	1.0098	(1.0068, 1.0129)
Family Practice Physicians			1.0011	(0.9969,1.0052)	1.0014	(0.9974, 1.0054)	1.0031	(0.9990, 1.0073)
Pediatricians			0.9959	(0.9900,1.0018)	0.9940	(0.9883, 0.9997)	1.0009	(0.9946, 1.0073)
Calendar year (ref=2015)								
2016			0.9671	(0.9423,0.9925)	0.9715	(0.9479, 0.9957)	1.0184	(0.9908, 1.0466)
2017			0.9145	(0.8881,0.9416)	0.9218	(0.8969, 0.9475)	0.9590	(0.9297, 0.9892)
2018			0.8918	(0.8616,0.9230)	0.8802	(0.8522, 0.9091)	0.9596	(0.9241, 0.9964)
2019			0.9177	(0.8825,0.9542)	0.8989	(0.8669, 0.9321)	0.9856	(0.9447, 1.0282)
2020			0.9979	(0.9570,1.0405)	1.0020	(0.9621, 1.0436)	1.1850	(1.1308, 1.2419)
Constant	0.2601	(0.2482,0.2725)	0.1117	(0.0970, 0.1286)	0.1285	(0.1122, 0.1471)	0.1195	(0.1004, 0.1422)
Number of child-year observations	432455		432455		432455		290523	

Note: Constant estimates baseline odds. Constant estimates baseline odds. 95% confidence intervals calculated using standard errors adjusted for two-way clustering by child 5-digit ZIP code and by child. [a] Sample is limited to children with at least one outpatient evaluation and management visit with any asthma diagnosis code in the calendar year.

© 2024 Geissler KH et al. *JAMA Network Open.*

eFigure 2. Unadjusted Probabilities of Receipt of Asthma Specialist Care

A. Unadjusted Probabilities of Receipt of Asthma Specialist Care by Insurance Type

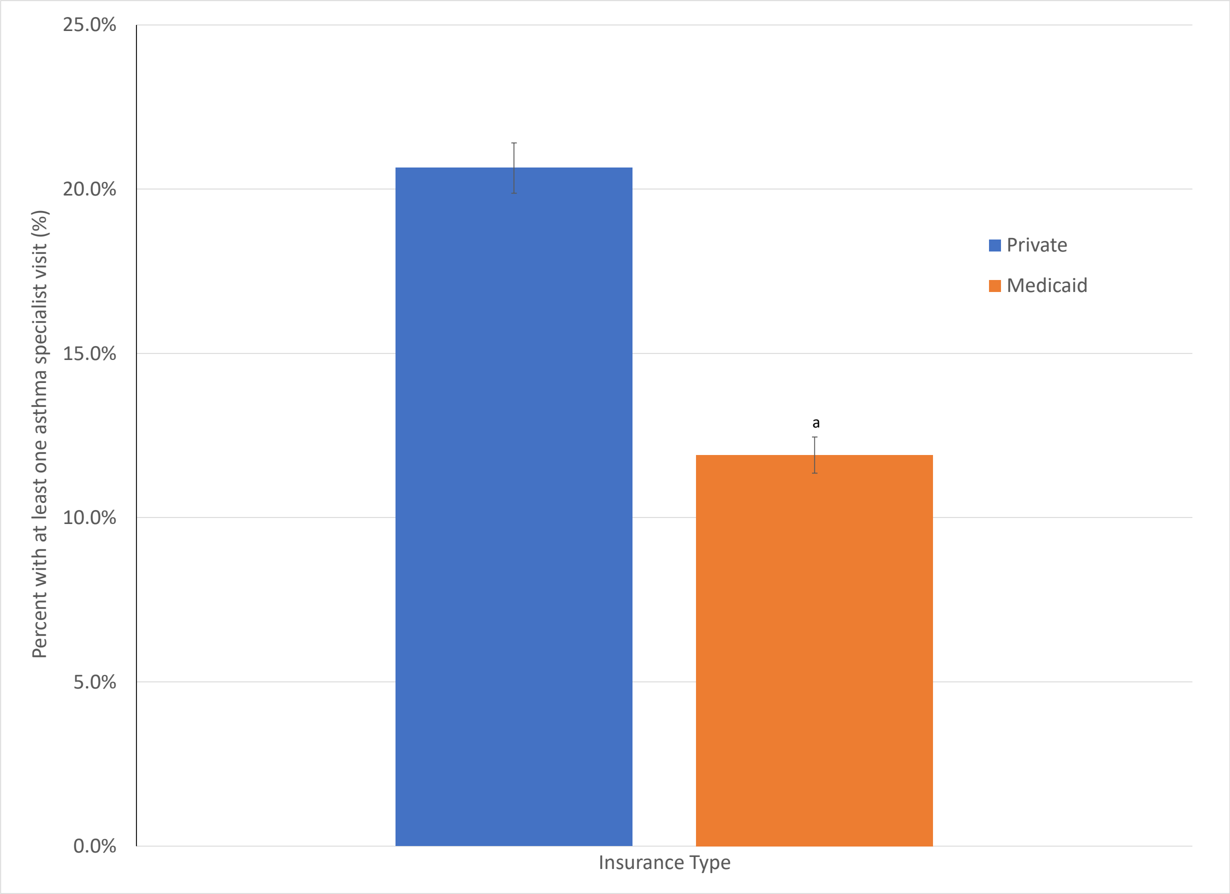

Note: [a] indicates difference between private and Medicaid is statistically significant at $p<0.05$. Predicted probabilities are calculated from a logistic regression without covariates. (N= 432,455)

© 2024 Geissler KH et al. *JAMA Network Open.*

B. Unadjusted Probabilities of Receipt of Asthma Specialist Care by Persistent Asthma

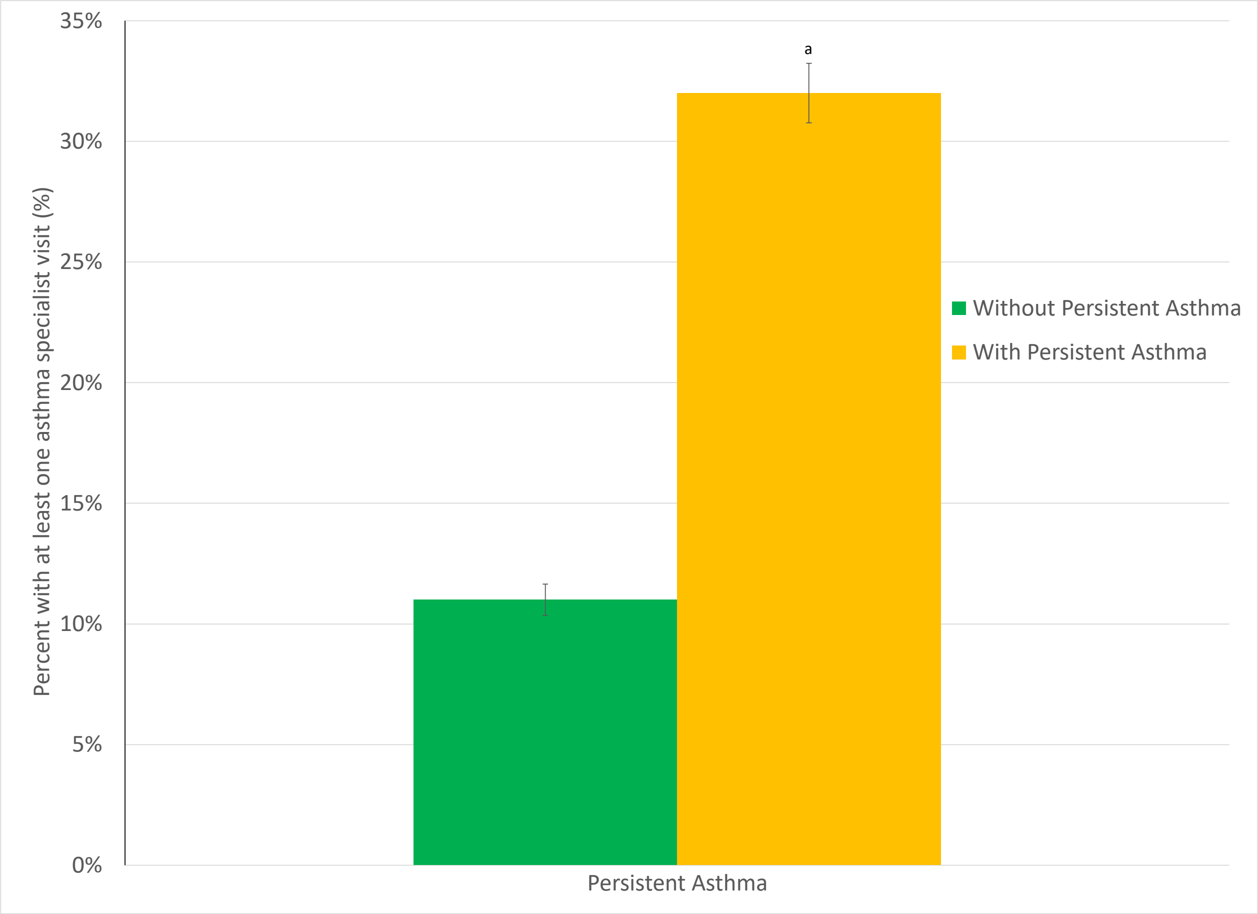

Note: [a] indicates difference between those with and without persistent asthma is statistically significant at p<0.05. Predicted probabilities are calculated from a logistic regression without covariates. (N= 432,455)

© 2024 Geissler KH et al. *JAMA Network Open.*

eFigure 3. Unadjusted Probabilities of Receipt of Asthma Specialist Care Over Time by Insurance Type

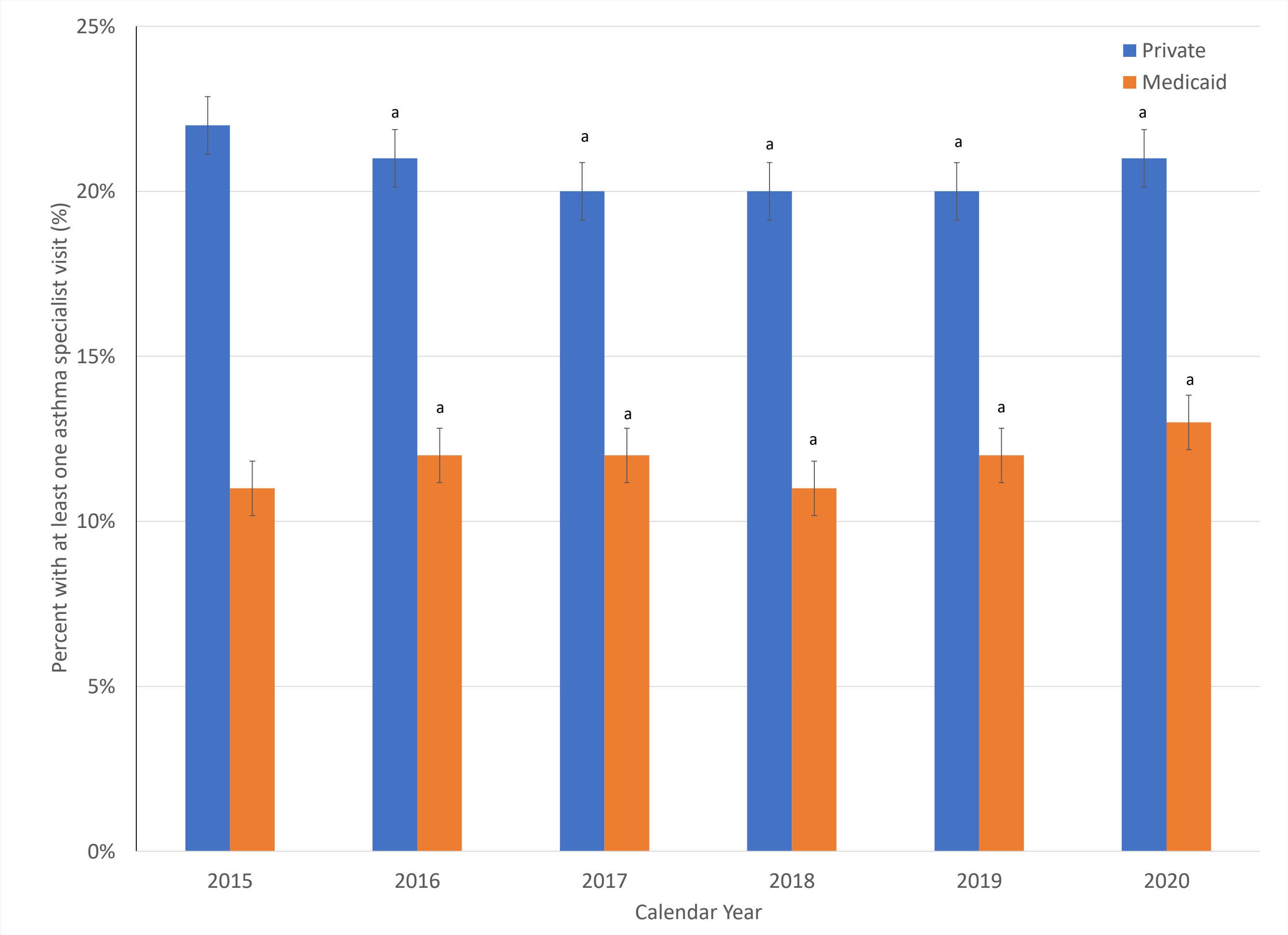

Note: [a] indicates difference from 2015 of same insurance type is statistically significant at $p<0.05$. Predicted probabilities are calculated from a logistic regression without covariates. (N= 432,455)

© 2024 Geissler KH et al. *JAMA Network Open.*

eFigure 4. Regression-Adjusted Predicted Probabilities of Receipt of Asthma Specialist Care Over Time Limited to Children With Persistent Asthma by Insurance Type

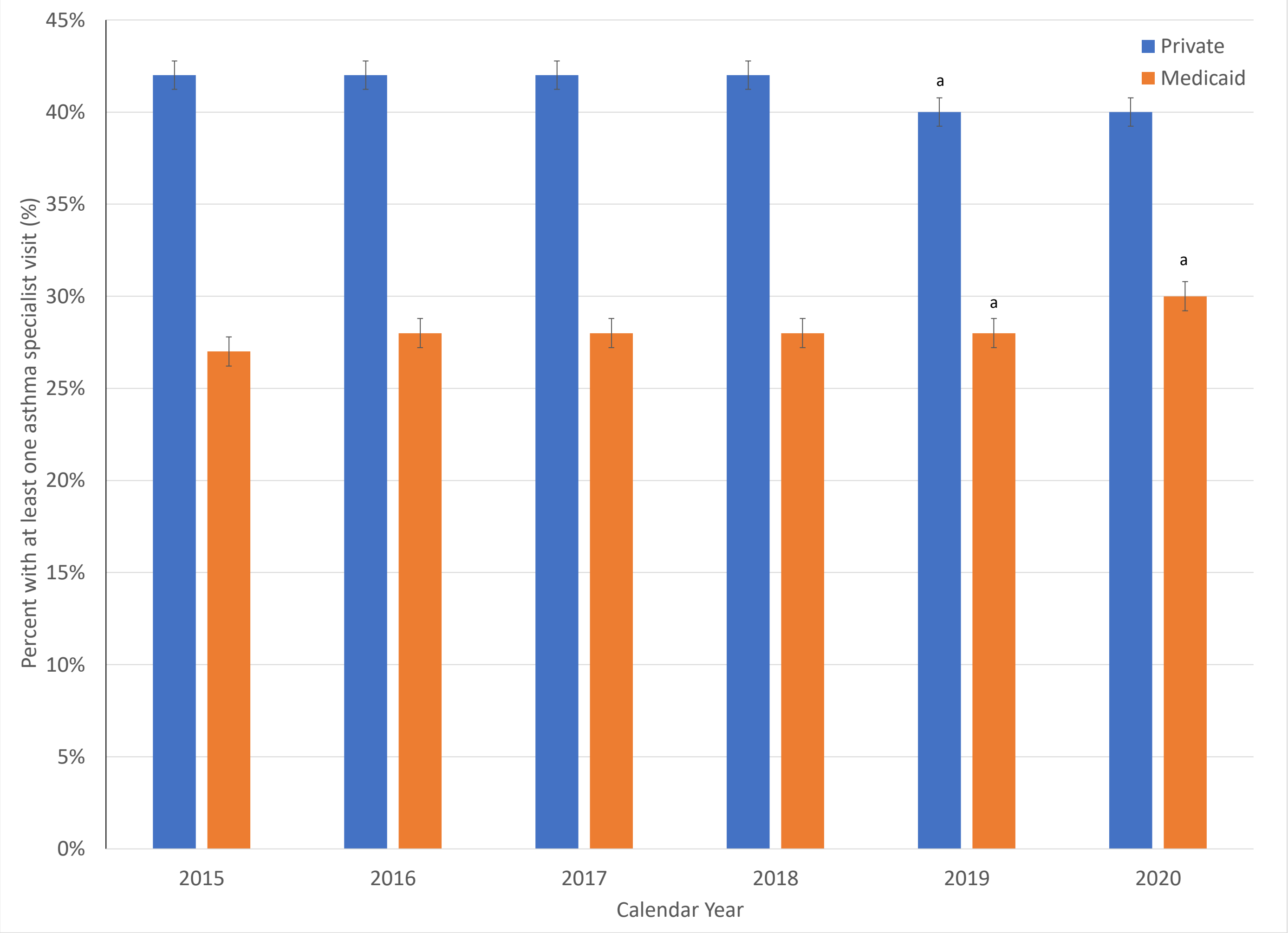

Note: [a] indicates difference from 2015 of same insurance type is statistically significant at p<0.05. Sample is limited to child-years with persistent asthma (N= 82,108). Predicted probabilities are calculated from a logistic regression adjusting for child demographics (age category and sex), DxCG risk score spline, indicator variables for calendar year, the structural racism effect index, and county level indicators of clinician supply (Nurse Practitioners, Physician Assistants, Family Medicine Physicians, and Pediatricians per 100,000 population).

© 2024 Geissler KH et al. *JAMA Network Open.*
