# Supplementary material for: Health Insurance Type and Outpatient Specialist Care Among Children With Asthma
Source: JAMA Netw Open. 2024 Jun 17;7(6):e2417319. doi: 10.1001/jamanetworkopen.2024.17319 (PMC11184461; doi:10.1001/jamanetworkopen.2024.17319)
Supplement: Supplement 2. — Data Sharing Statement [file jamanetwopen-e2417319-s002.pdf]

Data Sharing Statement

Geissler. Health Insurance Type and Outpatient Specialist Care Among Children With Asthma. *JAMA Netw Open*. Published June 17, 2024. doi:10.1001/jamanetworkopen.2024.17319

Data
Data available: No

Additional Information
Explanation for why data not available: Data obtained under restrictive data use agreement.
